# Female Rose Bitterling Prefer MHC-Dissimilar Males: Experimental Evidence

**DOI:** 10.1371/journal.pone.0040780

**Published:** 2012-07-18

**Authors:** Martin Reichard, Rowena Spence, Anna Bryjová, Josef Bryja, Carl Smith

**Affiliations:** 1 Institute of Vertebrate Biology, Academy of Sciences of the Czech Republic, Brno, Czech Republic; 2 School of Biology, University of St Andrews, St Andrews, United Kingdom; Utrecht University, The Netherlands

## Abstract

The role of genetic benefits in female mate choice remains a controversial aspect of sexual selection theory. In contrast to “good allele” models of sexual selection, “compatible allele” models of mate choice predict that females prefer mates with alleles complementary to their own rather than conferring additive effects. While correlative results suggest complementary genetic effects to be plausible, direct experimental evidence is scarce. A previous study on the Chinese rose bitterling (*Rhodeus ocellatus*) demonstrated a positive correlation between female mate choice, offspring growth and survival, and the functional dissimilarity between the Major Histocompatibility Complex (MHC) alleles of males and females. Here we directly tested whether females used cues associated with MHC genes to select genetically compatible males in an experimental framework. By sequentially pairing females with MHC similar and dissimilar males, based on *a priori* known MHC profiles, we showed that females discriminated between similar and dissimilar males and deposited significantly more eggs with MHC dissimilar males. Notably, the degree of dissimilarity was an important factor for female decision to mate, possibly indicating a potential threshold value of dissimilarity for decision making, or of an indirect effect of the MHC.

## Introduction

Genetic benefits of female mate choice are still a controversial aspect of sexual selection theory [1]–[3]. “Good genes” (more correctly “good allele”) models predict additive benefits of female choice, with some males representing a superior partner for all females in a population, leading to directional selection and often manifested by elaborate male signals [4]. “Compatible genes” (“compatible allele”) models predict that genetic benefits are non-additive and only a particular combination of male and female haplotypes can generate fitness benefits in the offspring [5]–[7], when parental alleles are complementary [8]. The mechanism for detecting mate compatibility is poorly understood, but the genes of the Major Histocompatibility Complex (MHC) have been proposed as a target of mate choice for compatible genes because of their unusually high variability [3], [9]. MHC genes primarily function in the vertebrate immune response, but it is suggested that MHC genes affect individual odor [10], [11], and thereby enable an individual to compare self and non-self odor, providing a potential mechanism underlying mate choice decisions for MHC compatibility [12], [13].

The high variability of MHC genes is generated by a coevolutionary arms race between the immune system and pathogens that impose selection on it. The adaptive value of high MHC diversity matches predictions from compatible genes models. The heterozygote advantage hypothesis [14] assumes overdominance effects; i.e. heterozygous individuals can mount an effective immune response to a broader spectrum of pathogens than homozygous individuals, with selection primarily favoring maximization of MHC diversity. However, in many taxa, the MHC is duplicated (or multiplicated), and optimal rather than maximum MHC variability may be favoured, due to negative T-cell selection during thymic development [15], [16]. The divergent allele hypothesis [17] predicts maximization of functional differences between alleles in heterozygous individuals, rather than MHC diversity *per se*, thereby increasing the range of pathogens targeted by immune system [18]. Finally, the negative frequency-dependent selection (rare allele advantage) hypothesis proposes rapid coevolution between hosts and pathogens, with rare alleles advantageous in pathogen resistance and with a dynamic turnover of beneficial alleles [19], which is consistent with a good genes model of mate choice.

A key prediction of MHC-based compatible allele models of mate choice is a mating preference for MHC-dissimilar or MHC-optimal mates. While evidence for MHC-based mate choice in natural populations is growing across several mammal, bird, reptile and fish mating systems [3], [20]–[22], it has so far been almost exclusively restricted to correlative results [23].

In a previous study [24] we demonstrated that female mating preferences in a small freshwater fish, the Chinese rose bitterling (*Rhodeus ocellatus*), matched basic predictions of compatible allele models of sexual selection. Female preferences were strong, but incongruent among individual females, and positively correlated with offspring survival and growth rate. There was a strong interaction of male and female genotype on offspring fitness, which also correlated with measures of mate MHC dissimilarity [24]. Unfortunately, Agbali et al. [24] compared their experimental outcomes with individual MHC profiles only after the tests were completed, yielding only a correlative outcome. Here, we expanded on this correlative result and designed an experimental study to directly test MHC-based mate choice in *R. ocellatus*. We tested whether female mating preference in *R. ocellatus* was predicted by MHC dissimilarity with potential mates, a central prediction of the hypothesis that females prefer males with compatible alleles to father their offspring. We tested this hypothesis in blind experimental trials by sequentially pairing female rose bitterling with MHC similar and dissimilar males, based on *a priori* known MHC profiles of individual fish, with the prediction that females would deposit more eggs with MHC dissimilar males.

## Materials and Methods

### Ethical Note

The study was conducted in accordance with Czech and British legal requirements. Experimental procedures were approved by the ethical committees of the Institute of Vertebrate Biology, Czech Academy of Sciences and Ministry of Agriculture (permits No. 3245/2003-1020, 065/2001-V1, 206/09/1163, CZ 62760203), and the University of St Andrews Animal Welfare and Ethics Committee. No collection or observation of fish was conducted in the field; the study was based solely on captive-bred fish. The target sample size was estimated based on previous comparable experiments to achieve a trade off between maximizing experimental power while minimizing the number of experimental fish used. Fish were housed in a UK Home Office approved facility in groups of 15 individuals in large 90 L aquaria with continuous aeration and filtration. Aquaria were enriched with a gravel substrate and artificial plants. Fish were returned to the same aquarium after experiments were completed, and retained as breeding stock. For pre-experimental and experimental conditions, see Experimental design.

### Study System

*Rhodeus ocellatus* lay their eggs in the gills of freshwater mussels where embryos are incubated for one month. During the reproductive period males establish territories around mussels and attract females to lay their eggs. Females lay multiple clutches each day and inspect both males and mussels prior to each decision to oviposit. There is no parental care; embryo development cannot be influenced by the parents once the eggs are laid. Female mate choice is strong in *R. ocellatus*, with female mating preference demonstrated by the decision to oviposit a clutch of eggs in a mussel guarded by a male [25] and positively related to size of this clutch (1–6 eggs) [26], [27]. Female mate preferences in *R. ocellatus* are based on olfactory cues, and weakly correlated with male courtship behavior [24], [28] There is no evidence for an effect of male size, dominance, or color in female mate choice decisions [25]. For a detailed description of the bitterling mating system, see [29], [30].

### Experimental Design

A total of 92 fish (27 females, 65 males) that were the second generation of a large outbred population of *R. ocellatus* originally imported from the River Yangtze basin, China, were individually marked using colored visible implant elastomer tags (VIE, Northwest Marine Technology company) and genotyped for MHC DAB1 and DAB3 alleles from a fin clip. Individual MHC profiles were identified for each male and female. For each female, one MHC similar and dissimilar male was assigned from the pool of genotyped males. MHC similarity/dissimilarity was maximized in terms of the functional differences between DAB1 alleles. We attempted to maximize contrasts between similar and dissimilar males by allocating the most similar and the most dissimilar male to particular female, given the constraints of our set of 65 genotyped males. A double blind approach was employed; genotyping (Brno, Czech Republic) and experimental tests (St Andrews, UK) were performed blind with respect to partner assignment as similar or dissimilar. Females were presented with males in sequential trials, with the order of male presentation (similar or dissimilar) randomized. Female mate preferences were measured sequentially rather than simultaneously to prevent a conflict between male-male competition and female preference [25]. One female died before the start of the experiment and six females failed to spawn during trials, resulting in a final sample size of 20 experimental females, paired with 40 males.

Experimental fish were housed in single sex groups in eight 54 L aquaria containing a gravel substrate and artificial plants. Mean (± s.e.) water temperature was 23.6 (±1.8) °C. Lighting was maintained on a 12∶12 h light:dark cycle. Fish were fed once daily with a mixture of frozen bloodworm and flake food. Female reproductive status was monitored each morning and those with ovulated eggs (recognized by extension of her ovipositor) were placed in a separate glass aquarium measuring 60 (length) x 30 (width) x 30 (depth) cm with a male (similar or dissimilar, see further) assigned according to experimental protocol. Experimental aquaria had a layer of sand as a substrate and a mussel in a sand-filled plastic box. Mussels were collected from the Grand Union Canal at Foxton, Leicestershire, and contained no bitterling eggs. After collection they were stored in an outdoor pond for 30 days before the start of the experiment. The pair was left to spawn for one hour. Any eggs deposited on the gills of the mussel were counted by gently prising the valves of the mussel open using a mussel opener. The female was subsequently paired with the second male assigned to her in another aquarium to avoid any carry-over of olfactory cues, and the number of eggs she deposited in a second, size-matched mussel was counted. Female preference was measured as the number of eggs deposited with each male. No fish or mussels were used again in the experiment.

### MHC Analysis

Genotyping of MHC focused on MHC Class II, which is known to be associated with mate choice in many vertebrate species. In most cyprinid fishes there is at least one functional gene (named DAB) encoding the MHC class IIβ chain of the protein [31]. This gene can be duplicated in cyprinid fishes, resulting in the expression of DAB1 and DAB3 genes [32]. In this study, we sequenced the complete (DAB1) or partial (DAB3) exon 2 encoding the β1 domain, which is the most polymorphic fragment of MHC Class II molecules responsible for antigen binding.

Genotyping, editing and alignment of MHC class IIB followed protocols described in [24]. In brief, we sequenced the complete exon 2 (encoding the most polymorphic fragment of MHC class IIB) using a combination of three primers located in introns and exon to minimize problems with null alleles. In addition to DAB1 sequences, we also sequenced the DAB3 gene (details below), which was only present in some individuals. There is no evidence of gene duplication at either DAB1 or DAB3 [5], [9]. DNA sequences were translated into amino acid sequences and those were used in all subsequent analyses.

For DAB1 alleles we examined selection on specific codons using the Random Effects Likelihood (REL) approach [33] implemented in the HyPhy software package [34]. Positively selected sites were considered those with a Bayes factor value >100 (15 out of 92 amino acid sites). A larger dataset of the present study resulted in higher Bayes factor estimates for positively selected sites compared to [24], though the position of positively selected sites remained unaffected. A Euclidean distance matrix of dissimilarity between alleles was calculated from functional differences between alleles using only positively selected sites [21], [24]. Functional differences between alleles are based on functional properties of individual amino acids (hydrophobicity, steric bulk, polarity and electronic effects) and describe differences in resulting proteins better than phylogenetic distances [21], [35].

Given that DAB3 alleles were only present in some individuals, we used two measures of MHC dissimilarity between partners. First, we adopted a maximum distance method, which used only the largest distance of all pairwise comparisons between the DAB1 alleles of two individuals [36]. This method does not consider the level of heterozygosity, but accounts for divergence between individual alleles by employing only the largest distance from all pairwise comparisons between male and female alleles (called ‘allele divergence’ henceforth). Second, we calculated the sum of all unique alleles across DAB1 and DAB3 loci of both parents. This measure is based on individual heterozygosity and comparable to the summation method of [37], [38]. This method does not measure allele divergence, but represents a simple sum of all unique DAB alleles across both parents and hence permits a combination of DAB1 and DAB3 alleles in a single matrix (called ‘allele summation’ henceforth). These two measures of divergence are not inherently correlated, although higher heterozygosity at DAB1 (higher estimate of ‘allele summation’) increases the likelihood of larger allele divergence from a larger number of possible combinations.

A total of 33 DAB1 alleles (92 amino acids long) were detected (File S1), with 17 alleles identical to alleles identified by Agbali et al. [24] for different individuals from the same experimental population. Heterozygote deficiency was observed, indicating the absence of the DAB1 locus on some chromosomes in our study population (10, 52 and 30 fish possessed no, single and two DAB1 alleles, respectively). Heterozygote deficiency resulted from copy number variation, with some chromosomes in the population lacking the DAB1 allele, rather than resulting from the existence of null alleles (see below for full details). Therefore, homozygote individuals were likely heterozygotes, with DAB1 present on one and absent on the second chromosome. Six DAB3 alleles (48 amino acids long) were identified (70, 21 and 1 fish possessed no, single and two DAB3 alleles, respectively). The MHC allele number is typically variable across individuals [3]. For example a fourfold (2–8) difference was identified in the three-spined stickleback (*Gasterosteus aculeatus*) [39].

Males with no DAB1 allele were treated as similar when paired with a female lacking DAB1 (2 cases) or possessing DAB1 alleles (three cases, always contrasted with a heterozygous male possessing alleles very dissimilar to the female’s alleles). Retrospectively, we acknowledge that our *a priori* assignment of three males with no DAB1 allele as being ‘similar’ to a female that possessed a DAB1 allele may be questionable, as it assumes that females perceive male dissimilarity based on the presence of a different allele (phenotypically expressed as a peptide), and hence base their choice on the presence of a different allele to their own (‘attraction’). However, this assignment is erroneous if female choice is based on a different mechanism, and a female possessing a DAB1 allele may perceive males with a different DAB1 allele as more similar than males with no DAB1 allele. Given this possibility, we ran all analysis with the complete (n = 20) and limited dataset (n = 17, excluding the three cases when a male with no DAB1 allele was treated as similar to a female with DAB1 present). The other five fish with no DAB1 were not used. Note that four out of seven fish with no DAB1 allele did possess DAB3 allele(s).

### Avoiding Null Alleles

Because of the highly polymorphic nature of MHC genes there is often a problem with null alleles; i.e. sequence variants that are not amplified because of substitutions in primer sites. To overcome this problem, we used two different strategies. First, the primers were designed in conservative parts of sequences on the basis of alignment of all available DAB sequences of cyprinid fishes. Second, the fragments of DAB genes were amplified using multiple combinations of primers located in various introns and exons (see below). If all possible combinations of primers failed to amplify the MHC fragment, the individual was considered as missing the particular DAB gene on both chromosomes. Poor quality of DNA extracts may also lead to failed PCR, so the quality of DNA was checked by PCR of microsatellite loci (for details on microsatellite amplification, see [25]); all fish with null DAB alleles amplified successfully. Therefore, we are confident that a lack of DAB3 alleles on many chromosomes reflects a real situation, rather than being an artifact of null alleles.

### DAB3 Genotyping Design

Two forward (DAB3-Ex1Fw and DAB3-Ex2Fw in exons 1 and 2, respectively) and two reverse (DAB3-Ex3rev and DAB3-Ex4rev1 in exons 3 and 4, respectively) primers were designed on the basis of homology with all available DAB3 sequences from cyprinid fishes downloaded from GenBank (Table 1). The specific amplification of partial sequences of DAB3 was checked by aligning the obtained sequences in BLAST. Three different PCRs were performed in the same conditions as for DAB1 (only annealing temperatures differed, Table 1). A total of six alleles was obtained (File S2). However, while all six alleles were successfully amplified by using the primer combinations II and III, only four alleles were amplified by combination I. This resulted in having only four sequences of partial (245 bp) exon 2. Our failure to obtain sufficiently long sequences of two DAB3 alleles, in combination with the fact that DAB3 alleles were expressed in a very low number of individuals, led us to use DAB3 data only in the ‘allele summation’ analysis. DAB3 sequences could not have been used in the ‘allele divergence’ analysis.

**Table 1:** PCR conditions and details on primers used for DAB3 sequencing.

Primer combination	Annealing temperature	Primer name	Primer sequence (5′- 3′)
I.	58°C	DAB3-Ex1Fw	CYCATACTGATGCTGTCTGC
		DAB3-Ex3rev	CCAGCTCAGAGTGAATCTGG
II.	59°C	DAB3-Ex2Fw	CAGCAGGTGAAAGCTCAGGTG
		DAB3-Ex3rev	CCAGCTCAGAGTGAATCTGG
III.	59°C	DAB3-Ex2Fw	CAGCAGGTGAAAGCTCAGGTG
		DAB3-Ex4rev1	GATGATTCCCAGCACCAGAC

### Sequencing and Cloning

All PCR products were purified by ExoSAP-IT® (USB) and directly sequenced using the BigDye Terminators Sequencing Kit v1.1 and an ABI PRISM 3130 Genetic Analyzer (Applied Biosystems). Homozygous sequences had no double peaks in electrophoretograms and they were confirmed by independent PCRs using at least two different sets of primers for DAB1 and two different DNA extractions for DAB3. Heterozygotes showed double peaks in variable sites and had to be cloned to separate individual alleles as described in [24]. The presence of PCR artifacts in cloned sequences (either substitutions or PCR recombinations; [40]) was checked by comparison with the heterozygous sequence obtained directly from genomic DNA.

### Checking for Pseudogenes

To avoid the possibility of analyzing pseudogenes, which can be common in MHC Class II in fish [31], we used two different approaches. First, we compared the genotypes of the DAB1 gene from six individuals obtained from complementary DNA (cDNA) and genomic DNA (gDNA). Total RNA was extracted from the spleen, stored in RNAlater (QIAGEN) by RNeasy Plus Mini Kit (QIAGEN) and cDNA was prepared by reverse transcription of 5 µg of total RNA by using SuperScript III reverse transcriptase (Invitrogen) and random hexamers as primers (Roche). Subsequently, we amplified exon 2 using primer combination III, sequenced purified PCR products and compared them with sequences obtained from genomic DNA. In all cases, the sequences of exon 2 obtained from RNA and DNA were identical, therefore providing evidence for transcription of the DAB1 gene.

Second, the sequences of exon 2 of both the DAB1 and DAB3 genes derived from *R. ocellatus* were edited and aligned in SeqScape v2.5 (Applied Biosystems). Aligned sequences were examined by eye for the presence of stop codons and/or insertions or deletions (‘indels’) causing a shift of the reading frame. None of them showed these types of mutation.

### Data Analysis

A general linear mixed model analysis (*nlme* package in R 2.9.1, LMM) was used to test the relationship between female preference (the number of eggs spawned) and male dissimilarity (‘allele divergence’ and ‘allele summation’). Maximal models included male dissimilarity, order of male presentation and their interaction as fixed factors, and female identity as a random factor. A second order effect was also tested for ‘allele summation’ to simulate a scenario of female choice for optimal rather than maximal heterozygosity. For each maximal model, model simplification was performed using a stepwise deletion procedure [41]. The non-significant terms were sequentially removed and likelihood ratio tests (using Maximum Likelihood) were employed to ensure that model simplification did not significantly reduce the amount of variance explained. This process was repeated until the minimal adequate model was obtained. Each removed term was then entered back into the minimal adequate model to obtain its level of non-significance. We used *a priori* selected measures of dissimilarity, chosen according to [24]. Given that our assignment of three experimental males as dissimilar was questioned during refereeing, we ran all analyses with the full dataset (n = 20 females, File S3) and limited dataset (n = 17 females, File S4). Further, to provide a complete overview of model selection, we additionally used the *MuMIn* package [42], [43] to evaluate all possible candidate models, including model averaging. This procedure yielded an identical outcome (File S5). Note that the use of dissimilarity measures based on the full sequences (92 amino acids) rather than positively selected sites had no effect on the outcome of the analysis.

## Results

**Figure 1:**
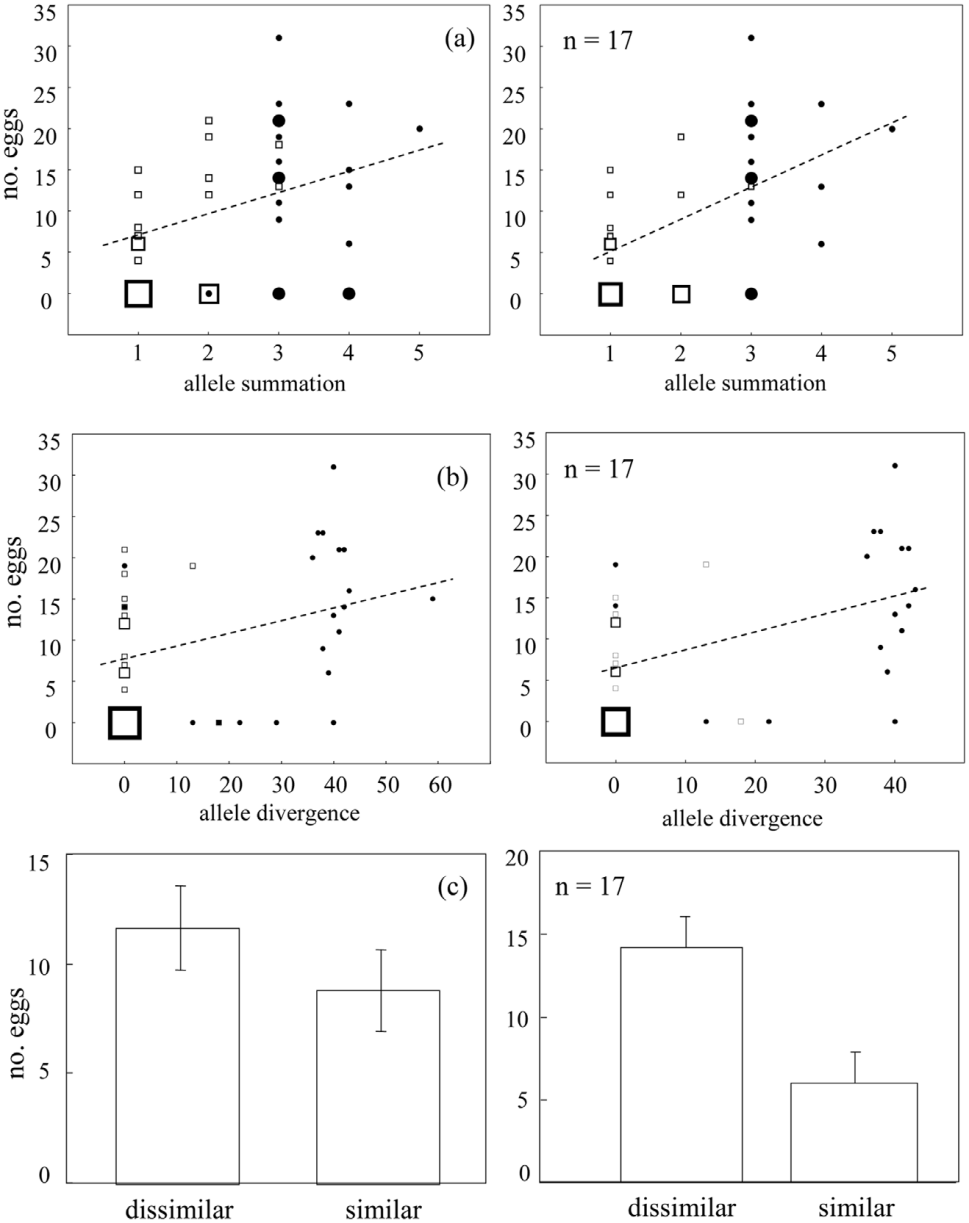
Relationship between measures of mate dissimilarity and female choice. The measures of mate dissimilarity were (a) allele summation, (b) allele divergence and (c) simple contrast. Full (n = 20, left panel) and limited (n = 17, right panel) datasets were used for calculation. Female choice was measured as the number of eggs deposited by females when spawning with a particular male. Symbol size (a, b) represents the level of overlap of individual data points, with a maximum of 6 overlapping points (the largest square). The line represents the best fit of a simple linear regression. Error bars (c) represent ±1 standard error.

Females discriminated between similar and dissimilar males and spawned significantly more eggs with MHC-dissimilar males. The outcome was robust across both dissimilarity measures used, with the magnitude of dissimilarity between male and female important for female mating decisions (Figure 1). Measures of ‘allele divergence’ and ‘allele summation’ were correlated (Pearson, r_19_ = 0.679, p = 0.001). For both measures, minimal adequate models contained only male dissimilarity (Table 2). Neither order of presentation nor the interaction between male dissimilarity and order of presentation improved the model (LR tests, all p>0.35). No effect of optimal number of alleles was detected (second order effect of ‘allele summation’; LR tests: p = 0.291 for n = 20 and p = 0.845 for a subset of n = 17). An analysis coding males simply as similar or dissimilar (excluding a quantitative measure of dissimilarity) demonstrated strong differences between the two male categories when the three cases with problematic partner assignment were excluded (n = 17, p = 0.005), though the trend was not statistically significant when the full dataset was used (n = 20, p = 0.072) (Table 2, Figure 1c).

**Table 2:** General Linear Mixed Models explaining female mate choice decision based on three measures of mate dissimilarity.

(a) Allele divergence		Full dataset (n = 20)					Reduced dataset (n = 17)			
Random Effects		Female ID 8.21					Female ID 8.21			
Fixed Effects	Estimate	SE	df	F	P	Estimate	SE	df	F	P
**(Intercept)**	**7.54**	**1.75**	**1,19**	**62.71**	**<0.001**	**6.27**	**1.81**	**1,16**	**57.71**	**<0.001**
**allele divergence**	**0.16**	**0.07**	**1,19**	**5.42**	**0.031**	**0.22**	**0.07**	**1,16**	**9.71**	**0.007**
order of presentation			1,18	0.66	0.428			1,15	0.08	0.786
interaction			1,17	<0.01	1.000			1,14	0.01	0.973
**(b) Allele summation**										
**Random Effects**		**Female ID 7.95**					**Female ID 7.95**			
**Fixed Effects**	**Estimate**	**SE**	**df**	**F**	**P**	**Estimate**	**SE**	**df**	**F**	**P**
**(Intercept)**	**3.35**	**3.11**	**1,19**	**63.55**	**<0.001**	0.72	3.02	1,16	60.59	0.812
**allele summation**	**2.83**	**1.15**	**1,19**	**6.00**	**0.024**	**4.03**	**1.17**	**1,16**	**11.8**	**0.003**
allele summation ^2			1,18	1.05	0.320			1,15	0.04	0.854
order of presentation			1,18	0.79	0.387			1,15	0.06	0.810
interaction			1,17	1.43	0.248			1,14	0.51	0.487
**(c) Similar vs. Dissimilar**										
**Random Effects**		**Female ID 8.66**					**Female ID 8.66**			
**Fixed Effects**	**Estimate**	**SE**	**df**	**F**	**P**	**Estimate**	**SE**	**df**	**F**	**P**
**(Intercept)**	**10.26**	**1.37**	**1,20**	**56.32**	**<0.001**	**14.2**	**1.89**	**1,16**	**57.3**	**<0.001**
**similarity**			1,19	3.63	0.072	**−8.18**	**2.67**	**1,16**	**9.41**	**0.009**
order of presentation			1,19	1.09	0.311			1,15	0.02	0.888
interaction			1,17	0.32	0.580			1,14	1.41	0.254

Table shows parameter estimates (with 1 standard error) for significant terms, degrees of freedom (df), test statistics (F), and significance levels (*P*). For the terms not included in the final model, the level of non-significance was obtained by entering the term into the minimal adequate model. The fixed terms included in minimal adequate models are indicated by bold typeset.

## Discussion

Confirmation that females from a range of taxa base their mate choice on cues associated with individual MHC profiles is accumulating, though evidence has so far chiefly been correlative. We used a direct experimental test of this hypothesis by contrasting the response of females to MHC-similar and MHC-dissimilar mates. Females spawned significantly more eggs with MHC-dissimilar males. Notably, the extent of dissimilarity was a significant factor. While we attempted to maximize the contrast between the two partners tested with each female, a lack of highly dissimilar males for some experimental replicates resulted in the use of males with intermediate dissimilarity. Interestingly, females did not lay eggs with these males (Figure 1b), suggesting that the level of mate dissimilarity is important and females are more likely to spawn with very dissimilar males. Fitness benefits of mate choice by female *R. ocellatus* arise through non-additive genetic effects [24], [25]. In this context, mate choice that takes advantage of a highly polymorphic genetic system, such as MHC, makes sense since these genes potentially enable individuals to avoid inbreeding and to select mates with complementary alleles. A mate choice system based on MHC also permits additive effects through preferences for specific MHC alleles, though there is no evidence for male additive genetic variance in the fitness of *R. ocellatus* [24].

The cues used by female *R. ocellatus* to detect individual MHC genotypes are not known, and may be directly or indirectly linked to MHC class IIB profiles. Similarity in individual odors is strongly related to similarity at the MHC [12], [13] and genes encoding odorant receptors are closely linked to the MHC [44]. In fish, MHC peptides on the cell surface of the olfactory receptors are likely involved in the process of odor recognition [11] and manipulation with MHC peptide ligands has been shown to affect female preference [45]. Individual odor cues are transmitted by body fluids, including urine [46] and possibly sperm [25]. In *R. ocellatus*, and many other fishes, courtship involves a quivering display, with the male undulating his body at high frequency, possibly to direct a current of water towards a female [24], followed by pre-oviposition sperm releases accompanied by female inspection of those cues prior to the decision to oviposit [30], [47]. It is notable, that the strength of female choice for dissimilar males was much higher when we excluded combinations where a male with no DAB1 allele was treated as similar to a female with a DAB1 allele present (and contrasted with dissimilar males possessing the DAB1 allele, different from that of the female). While this assignment was based on the assumption that female may detect odor cues containing a particular odorant, our data indicate that a female may perceive a lack of a specific odorant as more different than the presence of a dissimilar odorant. It is also possible that other functional alleles within the Major Histocompatibility Complex affect individual odors but are correlated with DAB alleles, or that odor cues only broadly correlate with MHC genotype. The question of whether individual odors serve as attractants or repellents during mate choice certainly deserves further investigation.

Our study demonstrated an association between MHC dissimilarity and female mate preferences, but selection may act only indirectly on the MHC genes and apparent female preference for MHC dissimilarity may only be a by-product of a preference for other functional loci that are linked to the MHC. Sherborne et al. [48] showed that mate choice patterns in house mice (*Mus musculus*) arose from dissimilarity in major urinary protein (MUP) genes, rather than the MHC, when the analysis controlled for the level of MHC similarity. The MUP are more stable than MHC-derived volatile compounds and hence appear to have more utility for scent marking in mice. Different taxa rely on other forms of communication (e.g. visual or auditory) and information on genetic identity could be encoded through alternative polymorphic mechanisms, which may or may not correlate with MHC [48], [49]. However, the opportunity for a compatibility-based mate choice system to evolve is likely hampered if many genes are involved and the interactions between genetic elements are complex. This is because complex genetic interactions will result in unpredictable offspring performance due to random segregation and crossing-over [50].

In some taxa, MHC genes are duplicated, with consequences for MHC-based mate choice. In *G. aculeatus*, an individual may express up to 9 individual alleles of MHC class IIB genes [16], compared to a maximum of 3 alleles expressed in *R. ocellatus* (present study). In *G. aculeatus*, females preferentially associated with odor cues from males with an optimal rather than maximal MHC heterozygosity [39], [45]. In contrast, we detected no evidence of female choice for optimal offspring heterozygosity in *R. ocellatus*. It appears that distinct mechanisms of MHC-based mate choice may operate in different taxa, possibly with optimization of offspring MHC diversity more frequent in taxa with multiplicated MHC genes and maximization of offspring MHC diversity in taxa with a limited MHC copy number. Further, maximization of offspring genetic dissimilarity may be adaptive for partners from the same population, while preference for intermediate offspring dissimilarity may be optimal for mate choice with a risk of outbreeding depression, for example when potential partners may come from different locally adapted populations and ecotypes [51], [52].

In summary, using an experimental approach with *a priori* defined treatment groups we found direct support for our previous correlative evidence that MHC dissimilarity matches mate choice decisions in *R. ocellatus*. While positive association between mate choice and offspring fitness has been experimentally demonstrated [24], our understanding of the role of MHC in mate choice would benefit from further insights, including expression of MHC-borne peptides in specific tissues, understanding of the mechanism of MHC signaling, and an insight into functional effect of a potential mating partners’ odor on mate choice decisions.

## Supporting Information

File S1 **Amino acid alignment for DAB1 alleles.**
(TXT)

File S2 **Amino acid alignment for DAB3 alleles.**
(TXT)

File S3 **Dataset used for statistical analyses with N = 20 females.**
(TXT)

File S4 **Dataset used for statistical analyses with a subset of N = 17 females.**
(TXT)

File S5 **A detailed overview of all candidate models using** ***MuMIn*** **package.**
(DOC)
