# Supplementary material for: Female Rose Bitterling Prefer MHC-Dissimilar Males: Experimental Evidence
Source: PLoS One. 2012 Jul 18;7(7):e40780. doi: 10.1371/journal.pone.0040780 (PMC3399850; doi:10.1371/journal.pone.0040780)
Supplement: File S5 — A detailed overview of all candidate models using MuMIn package. (DOC) [file pone.0040780.s005.doc]

**Supplementary Material, File S5: A detailed overview of all candidate models using *MuMIn* package**

**# NOTE THAT ALL ESTIMATES ARE BASED ON MAXIMUM LIKELIHOOD (MODEL COMPARISON), ESTIMATES SHOWN IN THE MAIN TEXT ARE BASED ON REML (PARAMETER ESTIMATES)**

**#Note that Allele divergence is called ‘diverg92aa’ in associated txt file, but ‘X92aa’ in the script, and Allele summation (Shared alleles) is called ‘summation’ in data file but ‘alT’ in the script.**

**# ALLELE DIVERGENCE, N = 20**

> dat<-read.table("c://MHC2010n20.txt",header=T)

> attach(dat)

> library(nlme)

> library(MuMIn)

> ad<-lme(eggs~X15aa*order,random=~1|female)

> ad<-lme(eggs~X15aa*order,random=~1|female,method="ML")

> AD<-dredge(ad)

> ADlist<-get.models(AD)

> list(AD)

[[1]]

Global model call: lme.formula(fixed = eggs ~ X15aa * order, random = ~1 | female,

method = "ML")

---

Model selection table

(Int) ord X15 ord:X15 df logLik AICc delta weight

**3 7.546 0.1585 4 -139.927 289.0 0.00 0.543**

**4 10.860 -2.130 0.1515 5 -139.575 290.9 1.92 0.208**

1 10.280 3 -142.594 291.9 2.86 0.130

2 14.550 -2.850 4 -142.030 293.2 4.21 0.066

8 10.870 -2.133 0.1513 0.0001622 6 -139.575 293.7 4.70 0.052

Random terms (all models):

‘1 | female’

> summary(model.avg(AD,subset=delta<2))

Call:

model.avg.model.selection(object = AD, subset = delta < 2)

Component models:

df logLik AICc Delta Weight

2 4 -139.93 289.00 0.00 0.72

12 5 -139.57 290.91 1.92 0.28

Term codes:

order X15aa

1 2

**Model-averaged coefficients:**

**Estimate Std. Error Adjusted SE z value Pr(>|z|)**

**(Intercept) 8.46443 3.14663 3.31393 2.554 0.0106 ***

**X15aa 0.15653 0.06837 0.07308 2.142 0.0322 ***

**order -2.13038 2.62726 2.81621 0.756 0.4494**

---

Signif. codes: 0 ‘***’ 0.001 ‘**’ 0.01 ‘*’ 0.05 ‘.’ 0.1 ‘ ’ 1

Full model-averaged coefficients (with shrinkage):

(Intercept) X15aa order

8.46443 0.15653 -0.59044

Relative variable importance:

(Intercept) order X15aa order:X15aa

1.00 0.28 1.00 0.00

>

**# SHARED ALLELES, N = 20**

sa<-lme(eggs~alT+order+alT:order+I(alT^2),random=~1|female,method="ML")

> Msubset<-expression(alT | !`I(alT^2)`) # the quadratic term can appear only together with linear term

> SA<-dredge(sa,subset=Msubset)

> SAlist<-get.models(SA)

> list(SA)

[[1]]

Global model call: lme.formula(fixed = eggs ~ alT + order + alT:order + I(alT^2),

random = ~1 | female, method = "ML")

---

Model selection table

(Int) alT alT^2 ord alT:ord df logLik AICc delta weight

**2 3.350 2.827 4 -139.662 288.5 0.00 0.388**

**4 -1.967 8.082 -1.0430 5 -139.104 290.0 1.51 0.183**

**6 7.031 2.734 -2.303 5 -139.241 290.2 1.78 0.159**

14 -5.826 7.847 5.194 -3.011 6 -138.461 291.5 3.00 0.087

1 10.280 3 -142.594 291.9 3.39 0.071

8 1.591 6.846 -0.8092 -1.481 6 -138.955 292.5 3.99 0.053

5 14.550 -2.850 4 -142.030 293.2 4.74 0.036

16 -8.548 10.300 -0.5676 5.133 -2.755 7 -138.320 294.1 5.67 0.023

Random terms (all models):

‘1 | female’

> summary(model.avg(SA,subset=**delta<2**))

Call:

model.avg.model.selection(object = SA, subset = delta < 2)

Component models:

df logLik AICc Delta Weight

1 4 -139.66 288.47 0.00 0.53

12 5 -139.10 289.97 1.51 0.25

13 5 -139.24 290.25 1.78 0.22

Term codes:

alT I(alT^2) order

1 2 3

Model-averaged coefficients:

Estimate Std. Error Adjusted SE z value Pr(>|z|)

(Intercept) 2.822 5.471 5.724 0.493 0.622

**alT 4.122 3.630 3.789 1.088 0.277**

**I(alT^2) -1.043 1.019 1.092 0.955 0.340**

**order -2.303 2.596 2.782 0.828 0.408**

Full model-averaged coefficients (with shrinkage):

(Intercept) alT I(alT^2) order

2.82232 4.12213 -0.26103 -0.50271

Relative variable importance:

(Intercept) alT I(alT^2) order alT:order

1.00 1.00 0.25 0.22 0.00

> # the model returns no significant term

> summary(model.avg(SA,subset=**delta<1.5**))

Call:

model.avg.model.selection(object = SA, subset = delta < 1.5)

Component models:

df logLik AICc Delta Weight

1 4 -139.66 288.47 0 1

Term codes:

alT

1

Model-averaged coefficients:

Estimate Std. Error Adjusted SE z value Pr(>|z|)

**(Intercept) 3.350 3.107 3.318 1.009 0.3127**

**alT 2.827 1.154 1.232 2.294 0.0218 ***

---

Signif. codes: 0 ‘***’ 0.001 ‘**’ 0.01 ‘*’ 0.05 ‘.’ 0.1 ‘ ’ 1

Full model-averaged coefficients (with shrinkage):

(Intercept) alT

3.3497 2.8267

Relative variable importance:

(Intercept) alT I(alT^2) order alT:order

1 1 0 0 0

>

**# PAIRED COMPARISON, N = 20**

> s<-lme(eggs~similarity*order,random=~1|female,method="ML")

> S<-dredge(s)

> Slist<-get.models(S)

> list(S)

[[1]]

Global model call: lme.formula(fixed = eggs ~ similarity * order, random = ~1 |

female, method = "ML")

---

Model selection table

(Int) ord sml ord:sml df logLik AICc delta weight

3 12.80 + 4 -140.769 290.7 0.00 0.422

1 10.28 3 -142.594 291.9 1.17 0.234

4 16.23 -2.3690 + 5 -140.348 292.5 1.78 0.173

2 14.55 -2.8500 4 -142.030 293.2 2.52 0.119

8 14.03 -0.8485 + + 6 -140.172 294.9 4.21 0.051

Random terms (all models):

‘1 | female’

> summary(model.avg(S,subset=delta<2))

Call:

model.avg.model.selection(object = S, subset = delta < 2)

Component models:

df logLik AICc Delta Weight

2 4 -140.77 290.68 0.00 0.51

(Null) 3 -142.59 291.85 1.17 0.28

12 5 -140.35 292.46 1.78 0.21

Term codes:

order similarity

1 2

Model-averaged coefficients:

Estimate Std. Error Adjusted SE z value Pr(>|z|)

**(Intercept) 12.803 3.233 3.364 3.806 0.000141 *****

**similarityS -4.981 2.658 2.842 1.753 0.079641 .**

**order -2.369 2.671 2.863 0.827 0.408069**

**---**

Signif. codes: 0 ‘***’ 0.001 ‘**’ 0.01 ‘*’ 0.05 ‘.’ 0.1 ‘ ’ 1

Full model-averaged coefficients (with shrinkage):

(Intercept) similarityS order

12.8032 -3.5729 -0.4945

Relative variable importance:

(Intercept) order similarity order:similarity

1.00 0.21 0.72 0.00

>

**# FOR A SUBSET OF REPLICATES WHERE MALES POSSESSING NO DAB1 ALLELE WERE NOT CONSIDERED SIMILAR TO FEMALES POSSESSING NO DAB1 ALLELE (MORE EXPLANATION IN THE MS TEXT)**

**# ALLELE DIVERGENCE, N = 17**

> dat<-read.table("c://**MHC2010n17.txt**",header=T)

> attach(dat)

> ad<-lme(eggs~X15aa*order,random=~1|female)

> ad<-lme(eggs~X15aa*order,random=~1|female,method="ML")

> AD<-dredge(ad)

> ADlist<-get.models(AD)

> list(AD)

[[1]]

Global model call: lme.formula(fixed = eggs ~ X15aa * order, random = ~1 | female,

method = "ML")

---

Model selection table

(Int) ord X15 ord:X15 df logLik AICc delta weight

**3 7.546 0.1585 4 -139.927 289.0 0.00 0.543**

**4 10.860 -2.130 0.1515 5 -139.575 290.9 1.92 0.208**

1 10.280 3 -142.594 291.9 2.86 0.130

2 14.550 -2.850 4 -142.030 293.2 4.21 0.066

8 10.870 -2.133 0.1513 0.0001622 6 -139.575 293.7 4.70 0.052

Random terms (all models):

‘1 | female’

> summary(model.avg(AD,subset=delta<2))

Call:

model.avg.model.selection(object = AD, subset = delta < 2)

Component models:

df logLik AICc Delta Weight

2 4 -139.93 289.00 0.00 0.72

12 5 -139.57 290.91 1.92 0.28

Term codes:

order X15aa

1 2

**Model-averaged coefficients:**

**Estimate Std. Error Adjusted SE z value Pr(>|z|)**

**(Intercept) 8.46443 3.14663 3.31393 2.554 0.0106 ***

**X15aa 0.15653 0.06837 0.07308 2.142 0.0322 ***

**order -2.13038 2.62726 2.81621 0.756 0.4494**

---

Signif. codes: 0 ‘***’ 0.001 ‘**’ 0.01 ‘*’ 0.05 ‘.’ 0.1 ‘ ’ 1

Full model-averaged coefficients (with shrinkage):

(Intercept) X15aa order

8.46443 0.15653 -0.59044

Relative variable importance:

(Intercept) order X15aa order:X15aa

1.00 0.28 1.00 0.00

>

**# SHARED ALLELES, N = 17**

> B<-lme(eggs~alT+order+alT:order+I(alT^2),random=~1|female,method="ML")

> Msubset<-expression(alT | !`I(alT^2)`) # the quadratic term can appear only together with linear term

> C<-dredge(B,subset=Msubset)

**> Clist<-get.models(C)**

> list(C)

[[1]]

Global model call: lme.formula(fixed = eggs ~ alT + order + alT:order + I(alT^2),

random = ~1 | female, method = "ML")

---

Model selection table

(Int) alT alT^2 ord alT:ord df logLik AICc delta weight

**2 0.7232 4.031 4 -115.978 241.3 0.00 0.591**

6 1.7890 3.991 -0.6496 5 -115.945 244.0 2.70 0.153

4 -0.1890 4.961 -0.1888 5 -115.959 244.1 2.73 0.151

14 -5.9750 7.201 3.8500 -1.889 6 -115.658 246.4 5.09 0.046

8 1.0870 4.525 -0.1069 -0.5367 6 -115.940 247.0 5.66 0.035

1 10.0900 3 -121.310 249.4 8.08 0.010

16 -5.5010 6.725 0.1232 3.9140 -1.970 7 -115.652 249.6 8.28 0.009

5 12.8200 -1.8240 4 -121.116 251.6 10.28 0.003

Random terms (all models):

‘1 | female’

**> > summary(model.avg(C,subset=delta<2))**

**Call:**

**model.avg.model.selection(object = C, subset = delta < 2)**

**Component models:**

**df logLik AICc Delta Weight**

**1 4 -115.98 241.34 0 1**

**Term codes:**

**alT**

**1**

**Model-averaged coefficients:**

**Estimate Std. Error Adjusted SE z value Pr(>|z|)**

**(Intercept) 0.7232 3.0199 3.2663 0.221 0.8248**

**alT 4.0305 1.1739 1.2697 3.174 0.0015 ****

**---**

**Signif. codes: 0 ‘***’ 0.001 ‘**’ 0.01 ‘*’ 0.05 ‘.’ 0.1 ‘ ’ 1**

**Full model-averaged coefficients (with shrinkage):**

**(Intercept) alT**

**0.72321 4.03052**

**Relative variable importance:**

**(Intercept) alT I(alT^2) order alT:order**

**1 1 0 0 0**

**# PAIRED COMPARISON, N = 17**

**> s<-lme(eggs~similarity*order,random=~1|female,method="ML")**

> S<-dredge(s)

> Slist<-get.models(S)

> list(S)

[[1]]

Global model call: lme.formula(fixed = eggs ~ similarity * order, random = ~1 |

female, method = "ML")

---

Model selection table

(Int) ord sml ord:sml df logLik AICc delta weight

3 14.18 + 4 -116.927 243.2 0.00 0.694

4 14.73 -0.3929 + 5 -116.916 246.0 2.74 0.176

8 10.14 2.8570 + + 6 -116.133 247.4 4.14 0.087

1 10.09 3 -121.310 249.4 6.19 0.032

2 12.82 -1.8240 4 -121.116 251.6 8.38 0.011

Random terms (all models):

‘1 | female’

**> summary(model.avg(S,subset=delta<2))**

**Call:**

**model.avg.model.selection(object = S, subset = delta < 2)**

**Component models:**

**df logLik AICc Delta Weight**

**1 4 -116.93 243.23 0 1**

**Term codes:**

**similarity**

**1**

**Model-averaged coefficients:**

**Estimate Std. Error Adjusted SE z value Pr(>|z|)**

**(Intercept) 14.176 1.885 2.039 6.954 < 2e-16 *****

**similarityS -8.176 2.665 2.883 2.836 0.00457 ****

**---**

**Signif. codes: 0 ‘***’ 0.001 ‘**’ 0.01 ‘*’ 0.05 ‘.’ 0.1 ‘ ’ 1**

**Full model-averaged coefficients (with shrinkage):**

**(Intercept) similarityS**

**14.1765 -8.1765**

**Relative variable importance:**

**(Intercept) order similarity order:similarity**

**1 0 1 0**

**>**
